# The Transmission Chain Analysis of 2014–2015 Ebola Virus Disease Outbreak in Koinadugu District, Sierra Leone: An Observational Study

**DOI:** 10.3389/fpubh.2017.00160

**Published:** 2017-07-10

**Authors:** Ifeanyi-Stanley Muoghalu, Francis Moses, Ishata Conteh, Patrick Swaray, Anthonia Ajudua, Anders Nordström

**Affiliations:** ^1^Institute of Tropical Medicine and International Health, Charité Universitätsmedizin, Berlin, Germany; ^2^World Health Organization (WHO), Koinadugu, Sierra Leone; ^3^District Health Management Health (DHMT), Koinadugu, Sierra Leone; ^4^African Union Support to EVD Outbreak in West Africa, The African Union Commission, Koinadugu, Sierra Leone; ^5^World Health Organization (WHO), Country Office, Freetown, Sierra Leone

**Keywords:** Ebola virus disease, transmission chain, outbreak response, epidemiology, public health intervention, community care center, Koinadugu, Sierra Leone

## Abstract

**Introduction:**

Sierra Leone experienced an unprecedented Ebola virus disease (EVD) outbreak in all its districts. Koinadugu District was the last to report an EVD case. Several outbreak response strategies were implemented. As part of lessons learnt, we conducted an observational study to describe the transmission chain in the district and the impact of the control measures implemented to contain the outbreak.

**Methods:**

We reconstructed the transmission chain, positioning both confirmed and probable cases, described the distribution of the EVD confirmed cases in the context of the routes of transmission (Community, Funeral or Health facility setting) and assessed the impact of control measures using the surveillance data collected during the outbreak.

**Results:**

All 142 confirmed and probable EVD cases registered were fully resolved in the transmission chain. 72.5% of all the EVD cases in the district were exposed in the community, 26.1% exposed during funerals, and 1.4% exposed in the health facility setting. Health-care workers contributed little to the EVD outbreak. 71.1% of EVD transmission occurred among family members. Female EVD cases generated more secondary cases than their male counterparts (*P* = 0.03). With removal of EVD cases from the community and admission to the community care center (CCC), the EVD transmission in the community decreased to substantially lower rates. In addition, transmission due to exposure in health facilities was further reduced with the implementation of full infection and prevention controls.

**Conclusion:**

This study details the transmission chain of EVD in a rural district setting and the public health interventions implemented to successfully limit the outbreak to just one of 11 chiefdoms. Heightened community-based surveillance for early case detection, swift isolation of suspect cases, efficient contact tracing and monitoring, and good infection prevention and control measures in health facilities were highly effective in limiting transmission and, eventually, breaking the transmission chain. CCCs were also instrumental in achieving early isolation and basic care for suspect cases, while ensuring that their family members who were close contacts remained in the community for easy contact tracing and monitoring. These were very useful lessons learnt that would inform the management of future outbreaks.

## Introduction

The 2014–2015 Ebola virus disease (EVD) outbreak in West Africa was unprecedented in nature and the largest documented EVD outbreak in the history of mankind (1, 2). The outbreak which started in the Southeastern forest region of Guinea in December 2013 was notified to the world on March 23, 2014, and by August 8, 2014, WHO declared the EVD outbreak a public health emergency of international concern (1, 2). It affected Guinea, Liberia, and Sierra Leone in a widespread manner. Other countries experienced local transmissions either through influx of infected travelers as in the case of Mali, Senegal, Nigeria, and USA or nosocomial transmission resulting from insufficient infection prevention and control (IPC) measures following medical evacuation as in the case of Spain, USA, and UK (1).

Ebola virus disease is an acute severe human infection caused by viruses of the family Filoviridae, including Ebolavirus, Marburgvirus, and Cuevavirus. Four species of Ebolavirus, namely, Zaire (ZEOV), Sudan (SUDV), Bundibugyo (BEOV), and Tai Forest (TAFV) (formerly Ivory Coast) have been associated with previous EVD outbreaks in humans (1, 3). Reston virus RESTV, another species of Ebolavirus, has been not associated with any EVD outbreak in humans (1, 3). Primary transmission of Ebolavirus is probably from contact with animal reservoirs, such as fruit bats or animals, notably non-human primates that have been infected by fruit bats. Secondary transmission is by person-to-person contact with body fluids of a symptomatic case which occurs mainly in three settings: in the family or community through contact with an infected individual or contaminated fomites, during funerals *via* touching or washing of dead bodies, and in the health-care setting due to poor infection and prevention control measures (4–6). Recent evidence has highlighted the possibility of sexual transmission from EVD survivors (7, 8). The incubation period ranges from 2 to 21 days. In previous EVD outbreaks, the case fatality rate (CFR) was around 50% but ranged from 25–90% (1, 3, 9).

Of the three countries with widespread transmission, Sierra Leone, a low-income country with a population of 7,092,113 million people (10), accounted for 57% (8,704/15,206) of total EVD-confirmed cases reported (1, 11). The first EVD case in Sierra Leone was confirmed on May 25, 2014, in Kailahun district bordering Guinea, and by October 2014, it had propagated to all 13 districts of Sierra Leone (12). Koinadugu district, the northernmost and largest district in Sierra Leone, was the last district to report an EVD-confirmed case on October 14, 2014.

During the course of the EVD outbreak in Koinadugu district, key response and control strategies were implemented. These strategies included early case detection and isolation of suspected EVD cases, active case finding for EVD suspected cases, contact tracing for EVD exposed persons, laboratory testing of samples (blood for most live EVD cases and saliva swab for dead EVD cases), case management for positive live EVD cases, quarantine for contacts of both live and deceased positive cases, social mobilization and community engagement, safe and dignified burials for EVD deceased, and coordination in line with the national EVD response plan. In addition, three Community Care Centers (CCCs) were established in remote areas of the EVD epicenter in Koinadugu district. The introduction of CCC was part of management of EVD in the community during EVD outbreak (13). Suspected and probable EVD patients were isolated and provided with basic health care at these CCCs while they awaited the confirmation of their samples collected. Positive cases were then transferred to Ebola treatment centers in other districts as there was no Ebola treatment centre (ETC) in Koinadugu district.

Since the end of EVD outbreak in Sierra Leone, there has been no detailed quantitative evaluation of the impact of these strategies on control of the EVD outbreak as part of lessons learnt in the Northern Province of Sierra Leone. The following questions remained unanswered:

- What was the quantification of the routes of transmission in Koinadugu district?
- What were the relative contributions of different settings to the spread of the EVD in the district?
- What was the effect of admission into the CCC on the transmission in the community?
- What were the impacts of the control measures instituted in the district?

With the information obtained from surveillance data and thorough field investigations, we reconstruct the chains of transmission to provide information about the origins of infection transmission as well as to describe the infection in different settings. As part of the post-EVD outbreak review, this paper describes the transmission chains of the EVD outbreak in Koinadugu District from October 2014 to April 2015. It also analyzes the transmission events in different settings that propagated the EVD outbreak in the district. It also describes the key time periods regulating the infection transmission. It assesses the effects of control measures on transmission during this period.

## Materials and Methods

### Study Design

This is a descriptive observational study conducted in Koinadugu district of Sierra Leone which is the largest district in Sierra Leone with a geographical area of 12,121 km^2^ accounting for just under one-fifth of the total size of Sierra Leone. It is bounded on the west, southwest, and southeast by Bombali, Tonkolili, and Kono districts, respectively, and shares a long international boundary with the Republic of Guinea in the North and Northeast. Koinadugu district is sparsely populated with a population of 408,075 (2015 National Population Census) of which 0–59 and 0–11 months are 72,233 and 16,324, respectively. It has 11 chiefdoms and 1,466 communities with 54.2% (794) hard-to-reach areas.

There are one secondary health-care facility (district hospital) and 74 primary health units (PHUs) in Koinadugu district. These 74 PHUs are categorized into 11 Community Health Centers, 21 Community Health Posts, 40 Maternal and Child Health Posts, and 2 non-governmental organization/private clinics. The District Health Management Team (DHMT) is responsible for ensuring that quality and equitable health services reach the population they serve.

### Data Source

We used all surveillance data collected during the EVD outbreak in Koinadugu district from October 2014 to November 2015. In addition, interviews from EVD cases and survivors, family, contacts, and community members during the EVD outbreak were used. During the EVD outbreak, WHO EVD outbreak standard case definitions for suspected, probable, and confirmed cases were used (14). The Ebola standard case investigation form was used to collect information on the line-list of suspected, probable, and confirmed cases. For the purpose of the study, we analyzed only the probable and confirmed cases. Suspected cases were excluded.

We used four complementary datasets generated during the outbreak. These were:

- the line-list of probable and confirmed cases
- the laboratory database
- the records from the CCC and holding center (HC)
- the results from additional epidemiological investigations for confirmed and probable cases

### Data Analysis

We analyzed the data using OpenEpi and prepared the transmission chain using Yed-graph Editor software.

### Outbreak

We considered both confirmed and probable cases. Data on age, sex, date of symptom onset, hospital admission, and death were obtained from the DHMT databases and the registers of the CCC and HC. We described the demographics of the EVD outbreak in Koinadugu district and the position of EVD probable and confirmed cases on the transmission chain.

### Transmission Chain

We reconstructed the transmission chain by analyzing the line-list, contact tracing forms, and transcripts of the interviews of EVD cases, EVD survivors, relatives of deceased EVD cases, extended family, contacts, and community members (the coauthors were involved in the outbreak management).

The initial transmission chain was discussed and agreed upon by the authors involved in the outbreak management. Information was further analyzed using the databases (containing the names of the contacts of primary cases, the relationships between primary case and contacts, the date of the last contact, and the place of contact exposure). We filled the gaps in the initial transmission chain by discussions with EVD survivors and relatives of deceased case patients. To this purpose, information obtained from the field epidemiological investigation notes of author ISM who visited all the villages affected in the district during the course of the outbreak was used to fill in information gaps when identifying any missing link. Furthermore, we explained events of transmission chains.

### Key Time Periods

We investigated the important periods regulating infection transmission by analyzing the complementary databases. We estimated the following:

(1) the incubation period, which is the time from exposure to symptom onset (it is computed as the time from the last contact with the infector to symptom onset);
(2) the time from symptom onset to admission at the CCC/HC/PHU, a measure of the transmission period in the community for isolated case patients;
(3) the time from symptom onset to death for non-hospitalized cases (the term non-hospitalized refers to EVD cases who died before or within 1 h after arriving at the CCC in Koinadugu district), a measure of the transmission period in the community for non-isolated cases;
(4) The serial interval, which is the time between symptom onset in a primary infector and in secondary cases. In this analysis, we discarded all missing data by assuming that they were missing at random.

We did not attempt to determine the time from hospitalization to discharge or death, which are indicative of the number of EVD beds required for timely isolation of cases. This is because once an EVD case was confirmed at the CCC, the case was immediately referred to the ETC. The travel time from the CCC or HC in Koinadugu to the ETC was also not determined as the data was not recorded. There were no ETCs in Koinadugu and all confirmed cases had to be referred to ETCs outside the district initially to Bo or Freetown, and later Bombali districts.

### Control Measures

During the EVD outbreak, three CCCs were constructed at the epicenter in order to isolate suspected or probable cases from the community. This is because the epicenter was in the hard-to-reach Nieni chiefdom, and it took a 10-h drive to get suspected or probable cases admitted into the Kabala Government Hospital (KGH) HC. In addition, IPC measures were fully implemented at all the isolation centers. Safe and dignified burials were done for deaths occurring in the community. We determined the effect of these measures on the transmission using the data collected.

### Ethics Clearance

The Sierra Leone Ethics and Scientific Review Committee and WHO Sierra Leone approved this Study protocol in September 2016. Because this study was based on data collected during surveillance and response activities of the EVD outbreak, informed content was not required. However, information on individual patients was anonymized before analysis for ethical reasons. Koinadugu DHMT permitted the use of the data for post-EVD outbreak evaluation.

## Results

### The Outbreak

**Figure 1:**
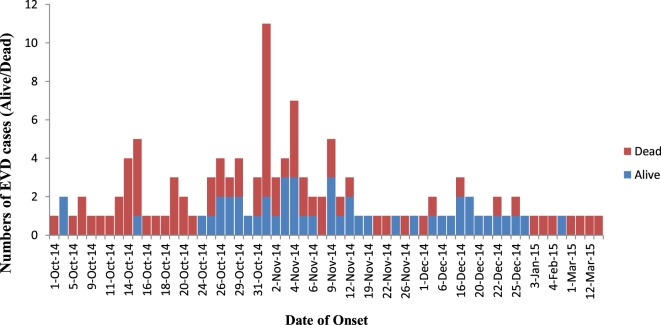
Epidemic curve of probable and confirmed cases of Ebola virus disease showing those alive and dead in Koinadugu District, Sierra Leone over time (October 2014 to April 2015).

In the study timeframe from October 2014 to April 2015, a total of 142 case patients, consisting of 111 confirmed and 31 probable cases were registered and positioned on the transmission chains in Koinadugu District (Table 1). Of these, 58 (40.8%) were male and 84 (59.2%) were female. Children (0–15 years) were 34 (23.9%) with mean age of 6.6 years (SD 4.5) while adults (16–85 years) were 108 (76.1%) with mean age of 40.6 years (SD 14.8). Overall mean age was 32.4 years (SD 19.6, Range 7 days–85 years). 2/142 (1.4%) were health workers. The overall CFR was 66.2% (94/142, 95% Cl 58.4–74.0). The CFR for children compared to that of adults were 64.7 and 66.7% (*P* = 0.41). However, the CFR for females compared to that of males were 73.8 and 55.2% (*P* = 0.01). Figure 1 shows the epidemic curve of EVD cases recorded in the district during the outbreak.

**Table 1:** Characteristics of probable and confirmed cases of Ebola virus disease in Koinadugu District, Sierra Leone from October 2014 to April 2015.

	Number, *n* = 142
Children (0–15 years old)	34 (23.9%)
Adult (16–85 years old)	108 (76.1%)
Mean age (years)^a^	32.4 (19.6)
Children	6.6 (4.5)
Adult	40.6 (14.8)
Females	84 (59.2%)
Health-care workers	2 (1.4%)
Confirmed cases	111 (78.2%)
Admitted to CCC, HC, or health facility	79 (55.6 %)
Death	94 (66.2 %)
Children vs adult	64.7 vs 66.7%; *p*-value = 0.41
Female vs male	73.8 vs 55.2%; *p*-value = 0.01
Community burials	15/62 (24.2%)
Cases imported into the district	4 (2.8%)
In the local transmission chains	138 (97.2%)
Exposure	
Possible infector	
Unknown	1 (0.7%)
1	134 (94.4%)
2 or more	7 (4.9%)
Place of Exposure	
Community	103 (72.5%)
Funeral	37 (26.1%)
Health facility	2 (1.4%)
Familial exposure: family/extended family	101 (71.1%)
Affected Chiefdoms	
Nieni	138 (97.2%)
Kasunko	1 (0.7%)
Wara Wara Yagala	3 (2.1%)

*^a^Data are presented in SD*.

### Transmission Chain

Throughout the duration of the EVD outbreak in Koinadugu District, four EVD cases entered the district. Only the first EVD case from neighboring Kono District in September 2014, resulted in the EVD outbreak experienced in Koinadugu district. The three other EVD cases which occurred late in December 2014 were from the Western Area and generated no secondary case.

The first imported case was a 70-year-old male resident of Kono district who had family relatives in Nieni chiefdom of Koinadugu district. After his exposure, he visited his first son in Fankoya community, Nieni chiefdom in Koinadugu district. Reportedly, he was never line-listed as a contact. Three days after his arrival, his illness worsened and he died in mid-September 2014. He became the origin of the transmission chain in Koinadugu district. Between mid-September and the end of September 2014, a total of 25 suspected EVD deaths comprising family and community members were reported to have occurred. On October 1, 2014, the DHMT received surveillance information about clusters of deaths in the hard-to-reach Nieni chiefdom. Nieni chiefdom accounted for the main outbreak with 97.2% (138/142) of the identified EVD cases. Kasunko and Wara-wara Yagala chiefdoms recorded one and three EVD cases respectively. The EVD cases were either imported or of unknown source.

**Figure 2:**
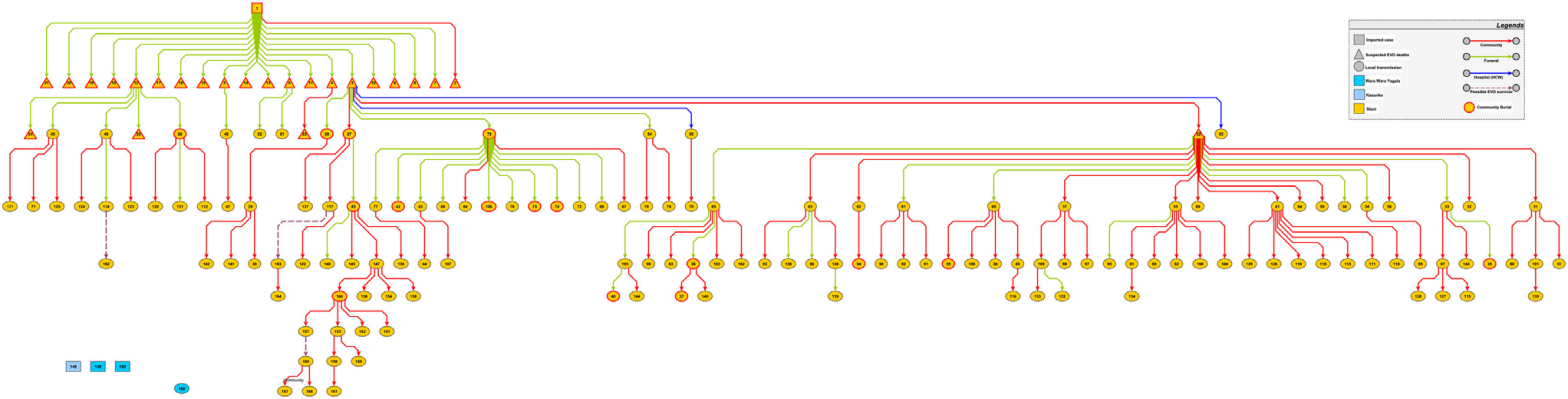
Transmission chains of Ebola virus disease outbreak in Koinadugu District, Sierra Leone (October 2014 to April 2015) symbols are defined in the figure and numbers are patients ID.

With the transmission tree well-resolved, 138/142 (97.2%) were involved in the local transmission (Figure 2). 134/142 (94.4%) cases had a single possible infector and 7/142 (4.9%) had two or more possible infectors. Only one case had no known infector and this was the last reported EVD case in the district (patient ID: 168).

**Figure 3:**
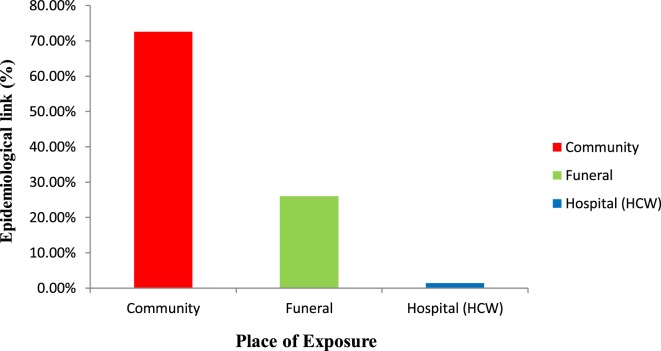
Context of transmission of Ebola virus disease in Koinadugu District, Sierra Leone (October 2014 to April 2015).

103 of 142 (72.5%) cases had contact exposure in the community with 37/142 (26.1%) exposed during funeral ceremonies and 2 (1.4%) cases exposed in a health facility (Figure 3). Specifically, the two cases were health-care workers (HCWs) (a facility In-charge and a vaccinator) at a PHU in the epicenter who attended to patients at the onset of the EVD outbreak before notification to the DHMT. Both HCWs died, resulting in a CFR of 100%. Toward the end of the transmission chains, 3 of 142 (2.1%) transmissions in the community were possibly linked to male EVD survivors (Patient IDs: 114, 117 and 157).

71.1% (101/142) of transmissions occurred between family members. 73/103 (70.9%: 95% Cl 61.6–79.0) of transmissions occurred between family members in the community and 28/37 (75.7%: 95% Cl 60.0–87.4) of those occurred at funerals.

**Figure 4:**
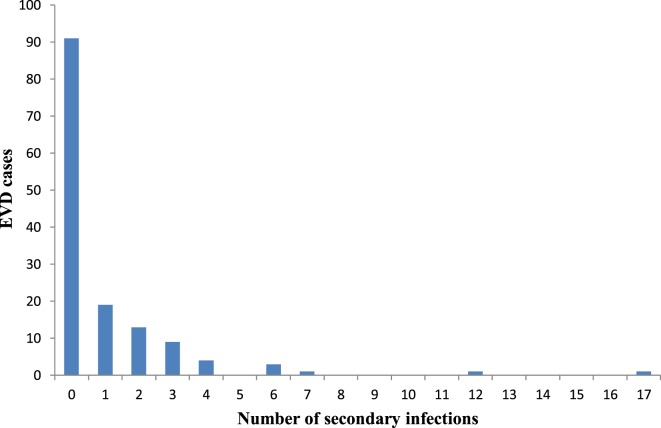
Distribution of number of secondary cases generated by Ebola virus disease cases in Koinadugu District, Sierra Leone.

49/142 (34.5%) cases sustained the chains of transmission generating secondary cases ranging from 1–17. Of those that generated secondary cases, 70.8% (34/49: 95% Cl 56.6–82.2) were female while 30.6% (15/49: 95% Cl 17.8–43.4) were male with a *P-value* of 0.03. Figure 4 shows the distribution of secondary cases. 91/142 (64.1%) did not transmit the infection. 19/142 (13.4%) generated only one secondary case; 13/142 (9.2%) generated two secondary cases. However, only 2 cases (patient IDs 22 and 73) generated more than 10 secondary cases which were 17 and 12, respectively. These two patients were well-known for washing dead bodies and were exposed in Fankoya community. When patient ID 22 was sick, her son (ID: 58) took her to three different communities in search of care. In the first community Sumbaria, a traditional healer (ID: 57) attended to her for 2 days. Employing the services of a motorcycle rider (ID: 41) from another community Kendeya, she was taken by her son to a religious house in another community Kumala where she was cared for by nine women for 3 days (patient ID: 31, 32, 55, 56, 59, 61, 62, 63, and 64). After unsuccessful traditional and religious treatment, she was taken back to her place of birth in Yoria community where, she died a few minutes after arrival and a community burial was done. Patient ID 73 became sick in her husband’s home in Liro village and she infected four family members (ID: 42, 84, 86, and 106) who cared for her. As her illness worsened, her brother (ID: 72) who resided in another community—Funubakura, took her to his home where she infected other eight family members (ID: 43, 67, 68, 74, 75, 76, and 77).

**Figure 5:**
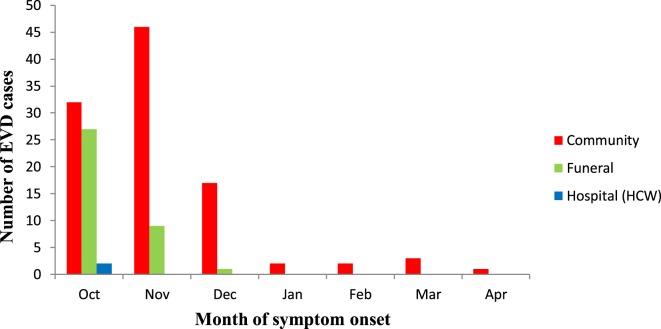
Distribution of number of Ebola virus disease cases by month of symptom onset in Koinadugu District, Sierra Leone (October 2014 to April 2015).

Analysis of the number of cases by month of onset showed that 61 of 142 (43.0%) were reported in October while 38.7% (55/142) and 12.7% (18/142) of cases were reported in November and December 2014, respectively. 2/142 (1.4%) cases were reported in January 2015 and in February 2015. It slightly increased to 3/142 (2.1%) in March 2015. In April 2015, only 1 (0.7%) EVD case was reported. When stratified by the place of contact exposure, an increase of 44% in the number of cases was shown, due to exposure in the community from October (32) to November (46) 2014. During the same period, however, the cases due to exposure during funerals decreased by 67%. Only in October 2014, 2 of 61 (3.3%) cases occurred due to exposure in the health facility (Figure 5). HCWs contributed little to the spread of the EVD outbreak. Subsequently over time, the number of infections decreased substantially.

### Key Time Periods

The mean incubation period was 9.5 days (range, 3–18 days). The mean time of symptom onset to admission into the health facility was 5.1 (range, 1–20 days). The mean time of symptom onset to death for non-admitted cases in the community was 6.7 (range, 0–15 days). The mean serial interval was estimated to be 11.1 days (range, 2–19). Table 2 summarized the estimates of the natural history.

**Table 2:** Estimated key time periods (mean, median, SD, range) and number of observations (*N*) of Ebola virus disease (EVD) outbreak in Koinadugu district, Sierra Leone.

Estimated key time periods (mean, median, SD, range)	*N*	Mean	SD	Range
Incubation period	76	9.5	4.0	3–18
Onset of symptom to admission at CCC, KHC, or CHC	76	5.1	4.0	0–20
Onset of symptom to death in community	27	6.7	4.1	0–15
Serial interval^a^	47	11.1	5.2	2–19

*^a^Excludes the serial interval for the possible EVD survivors*.

### Control Measures

**Figure 6:**
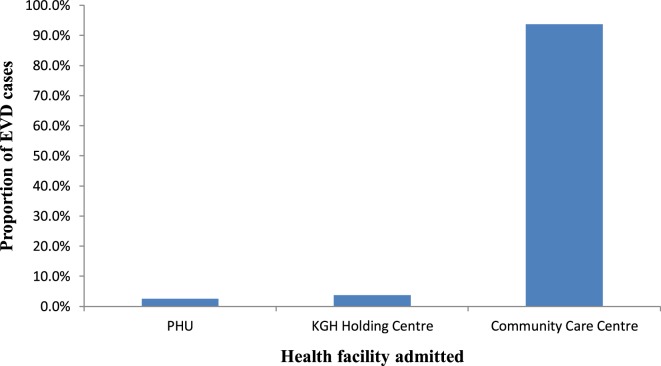
Proportion of Ebola virus disease cases admitted into health facility in Koinadugu District, Sierra Leone.

Of the three CCCs constructed in the district, only one CCC was used for isolation of suspected or probable EVD cases pending confirmation. The proportion of admitted cases was 55.6% (79/142). Among the admitted cases, 2.5% (2/79) were admitted into PHU, 3.8% (3/79) admitted at the KGH HC and 93.7% (74/79) admitted at the CCC (Figure 6). Admission into the PHU occurred at the beginning of the outbreak before the construction of CCC. By admitting cases into the CCC from the community, the transmission reduced by 63 to 88% between November 2014 and January 2015, respectively. After establishment of the CCC and institution of full IPC measures, the transmission due to exposure in the health facility reduced to 0%.

Non-admitted cases constituted 44.4% (63/142). Apart from 1 (1.6%) case who died on transit to the KGH HC, 98.4% (62/63) died in the community. Among deaths in the community, 24.2% (15/62) had community burials. 60% of community burials occurred in October, 33.3% in November and 16.7% in December 2014. Safe and dignified burials accounted for 75.8% (47/62). With the implementation of safe and dignified burials, the transmission due to exposure during funerals decreased by 89% between November and December 2014, and by 100% through to the end of the outbreak.

## Discussion

The EVD outbreak in Sierra Leone was intense and affected all the districts. Prior to this, there was no analysis of the EVD transmission in the Northern Province of Sierra Leone. As part of post-epidemic evaluation, this detailed quantification of the EVD outbreak in Koinadugu district provides a unique opportunity to understand the transmission of EVD in a rural district of Sierra Leone.

The findings of the study show that most transmissions took place in the community and between family members. However, these transmissions within community decreased to substantially low rates once isolation into the CCC was implemented. Transmission during funerals contributed a little after the safe dignified burials were put in place. Although transmission due to exposure at the health facility had a minor role to the spread of the outbreak in the district, the full implementation of IPC measures at the health facilities further reduced the chances of transmission. Furthermore, our study revealed that higher numbers of secondary EVD cases were generated from female primary EVD cases than their male counterparts and was statistically significant.

The high transmission of EVD cases within family members from our study was congruent with other studies in Pujehun district of Sierra Leone (15) and in Conakry prefecture of Guinea (16) which reported high transmission within family members of 74.3 and 72%, respectively. This is not unrelated to the complexity in care of the sick within the African context as described by Borchert and colleagues in the outbreak of Masindi district Uganda (4). Similar to the study in Conakry Guinea (16), more transmission of EVD occurred in the community. Conversely, like the Pujehun study (15), transmission due to exposure in the health facility was much lower than that reported in the urban Conakry study (16). This is likely to be a reflection of the category of the health facilities.

Koinadugu district, being the last district to report EVD case, had already set up some measures such as checkpoints and screening stations on the only tarmac access road leading into the district in order to minimize movement of people into the district. Other measures such as social mobilization and community awareness-raising campaigns about the ongoing EVD outbreak and methods of prevention were perceived to be strong within the district capital town and its environs. This led to institution of by-laws by local authorities (Paramount Chiefs) instructing community members to report any new visitors to their communities to local authorities and health personnel, as well as to report all deaths and allow safe and dignified burials of the deceased by a trained burial team. Violations of these laws were penalized by heavy fines. However, hard-to-reach communities benefited less from the social mobilization and community engagement activities due to rough terrain and long driving hours. In addition, there was initially little or no training of health workers on EVD case definitions. This asymmetric knowledge across the district resulted to the first imported EVD case from a neighboring district Kono becoming the origin of the EVD outbreak. This case did not use the major access road which was the focus of attention in the EVD response by the District Ebola Task Force team. After the first introduction of EVD into the district, there were heightened EVD surveillance activities such as training of health workers and community health workers on the standard case definition for EVD with emphasis on history of recent travel to EVD-affected areas, early detection and immediately reporting. In addition, there was engagement of the community leaders in order to increase community acceptance toward the EVD response workers. Due to high popularity of activities of the traditional healers in the district, there was also training of traditional healers focused on early detection and immediately reporting of any suspected EVD cases. These actions may have most likely contributed to prevention of subsequent EVD outbreaks in other parts of the district as in the case of the three other imported EVD cases which did not generate any secondary cases. Interestingly, these other EVD imported cases which occurred in late December 2014 were reported early to the EVD response team and isolated into KGH HC within 24-h of arrival into the district.

Another interesting transmission source reported in our study was the possibility of transmission from male EVD survivors. Although there was no genetic sequencing test at the time of field investigation to confirm this source of transmission, this assumption was most plausible after exclusion of other potential sources of transmission. Notably, there were certain similarities among these three EVD cases. First, there was no active transmission chain in the community of residence at the time the three different cases were investigated and reported. Second, the EVD cases were female spouses of male EVD survivors who either cohabitated or visited and spent time after they were discharged from ETC. Third, there was collaborative narrative from the male EVD survivors acknowledging co-habitation. Finally, there was restriction of movement of persons as part of the EVD strategy instituted by National Ebola Response Centre. The other argument for this source of transmission is that it might be cryptic or isolated transmission. In order to resolve this dilemma in future EVD outbreaks, the use of genetic sequencing tests will be needed to complement field investigation in identification of the transmission source.

In Koinadugu district, the EVD cases occurring at the health facility setting involved the only two HCWs and resulted in a CFR of 100%. Although this occurred early in the outbreak, it reflected the inadequate surveillance preparedness and poor knowledge of IPC among health workers in Sierra Leone (6). Furthermore, the EVD outbreak also affected the district healthcare system resulting in the closure of two health facilities within the epicenter, Nieni Chiefdom of Koinadugu district, thereby revealing the challenges in emergency preparedness.

The CCC was useful in Koinadugu district as it contributed to the prevention of movement of suspect or probable cases from the epicenter to the district’s only hospital where a temporary HC had been established. The importance of CCC is in line with the report from Sierra Leone experience by Olushayo Olu and colleagues that highlighted the CCC as an adjunct innovative response to the containment of the EVD transmission and the care of those affected (17). In the Koinadugu context, the CCC aimed to address two unique challenges. First, movement of EVD cases to the hospital, located more than 150 km away from the epicenter, was impracticable due to poor roads and muddy terrain, resulting in ambulances getting stuck along the way. The first few patients had to spend more than 24 h in the ambulance. Second, a major challenge posed by movement of suspect and probable cases was that close family members, who were the most likely contacts if the patient was a positive case, would want to follow. This would have made contact tracing more complicated and exposed people living in the communities around the hospital to the risk of contacts becoming symptomatic while living among them.

### Study Limitations

Incompleteness of data and poor data quality of the databases contributed to the limitations of this study. Missing variables such as date of onset especially with the initial cases existed in the databases and this was challenging. In other instances, the contact tracing database was not available due to unexpected corruption of the dataset. It is important to understand that data collection was done under extremely difficult conditions. However, we addressed this challenge by cleaning the database and excluding records with missing variables.

Another limitation to this study was the challenge posed in the reconstruction of transmission chains. We attempted to resolve the chains of transmissions of EVD in Koinadugu district. Field epidemiologists involved in the outbreak synthesized all the epidemiological data and their field notes; for each case, we agreed on the most probable sources of infection and contexts of infection.

## Conclusion

This study has described the transmission chain of EVD in a rural district of Northern Sierra Leone, one of the hardest hit countries during the 2014–2015 EVD outbreak. In general, the public health interventions implemented in the district seems to have contained the outbreak in just one of 11 chiefdoms. Three sporadic cases occurring in other parts of the district later in the outbreak were prevented from generating secondary cases due to the implementation of swift and effective public health measures including heightened community-based surveillance for early detection, swift isolation of suspect cases, efficient contact tracing and monitoring, and good IPC measures. Decentralization of isolation centers by setting up CCCs ensured that EVD cases were removed early from communities, breaking the transmission chains within the community. This strategy also helped ensure that family members of positive cases, who were close contacts, did not have to move from the epicenter of the outbreak to follow their relatives to a distant point of care. This meant that contact tracing and monitoring was easier as contacts were less likely to travel. These public health interventions proved effective in limiting transmission in Koinadugu and would be very useful as lessons learnt for the management of any future outbreaks.

## Author Notes

Ifeanyi-Stanley Muoghalu worked as a consultant epidemiologist to support World Health Organization Ebola Response Team, WHO headquarters in Geneva. Ifeanyi-Stanley Muoghalu was deployed to Sierra Leone as field epidemiologist to support WHO response to EVD outbreak in districts from December 2014 to March 2016. Francis Moses is district medial officer of Koinadugu District Health Management Team. Ishata Conteh was staff of WHO Sierra Leone and worked as a field epidemiologist in various districts. Patrick Swaray worked in consultancy capacity as a Contact Tracing Supervisor with WHO in Koinadugu District. Anthonia Ajudua was deployed as field epidemiologist with the African Union support to EVD outbreak in West Africa. Anders Nordström is the WHO Representative in Sierra Leone. The views expressed in this article are those of the authors alone and do not necessarily represent the views, decisions, or policies of the institutions with which they are affiliated.

## Author Contributions

I-SM and FM designed and coordinated the study. I-SM, FM, IC, PS, and AA were involved in the outbreak investigation and collected the data. I-SM analyzed the data. I-SM, FM, IC, PS, and AA contributed in interpreting the results. I-SM and FM drafted the Manuscript. All authors read and provided input in the final draft of the manuscript and agreed to be accountable for all aspects of the work. FM and AN gave final approval for the publication of the manuscript.

## Conflict of Interest Statement

The authors declare that the research was conducted in the absence of any commercial or financial relationships that could be construed as a potential conflict of interest.
